# Public Awareness and Knowledge of Oral Cancer in 13 Middle Eastern and North African Countries

**DOI:** 10.1001/jamanetworkopen.2025.0522

**Published:** 2025-03-06

**Authors:** Mohammad Zakaria Nassani, Anas Alsalhani, Faisal Mehsen Alali, Samer Rastam, Nasser Raqe Alqhtani, Abdullah Saad Alqahtahni, Ali Robaian, Faisal S. Alhedyan, Abdullah Bin Nabhan, Adel Alenazi, Khalid Ayidh Alqahtani, Ali Alrafedah, Abdullah Ahmed Abbas Alleft, Banna Alnufaiy, Rafif Alshenaiber, Rawda Omar Alghabban, Maram Alagla, Mohammed A. S. Abuelqomsan, Maya Al-Joukhadar, Noujoud Al Zahed, Shorouk Darwish, Azza Sioufi, Enass Shamsy, Omar Kujan, Mohammed Noushad, Sadeq Ali Al-Maweri, Abdulaziz Binrayes, Basem Sabbagh, Bassel Tarakji

**Affiliations:** 1 Department of Restorative and Prosthetic Dental Sciences, College of Dentistry, Dar Al Uloom University, Riyadh, Saudi Arabia; 2 Department of Dentistry, Vision Colleges, Riyadh, Saudi Arabia; 3 Department of Oral and Maxillofacial Surgery and Diagnostic Sciences, College of Dentistry, Prince Sattam bin Abdulaziz University, Al-Kharj, Saudi Arabia; 4 Department of Medicine, Vision Colleges in Riyadh, Riyadh, Saudi Arabia; 5 Department of Preventive Dental Sciences, College of Dentistry, Prince Sattam bin Abdulaziz University, Al-Kharj, Saudi Arabia; 6 Implant and Restorative Dentistry, Department of Conservative Dental Science, College of Dentistry, Prince Sattam bin Abdulaziz University, Al-Kharj, Kingdom of Saudi Arabia; 7 Department of Histology and Pathology, Faculty of Dentistry, University of Hama, Hama, Syria; 8 Department of Prosthetic Dental Sciences, Prince Sattam bin Abdulaziz University, Al-Kharj, Saudi Arabia; 9 Department of Pediatric Dental Sciences, College of Dentistry, Prince Sattam bin Abdulaziz University, Al-Kharj, Saudi Arabia; 10 Department of Conservative Dental Science, College of Dentistry, Prince Sattam bin Abdulaziz University, Al-Kharj, Kingdom of Saudi Arabia; 11 Department of Oral Histology and Pathology, College of Dentistry, Arab International University, Damascus, Syria; 12 Cellular and Molecular Biology, Faculty of Sciences Branch III, Lebanese University, Tripoli, Lebanon; 13 Ministry of Health, Damascus, Syria; 14 Aljazeera Hospital, Riyadh, Saudi Arabia; 15 Department of Restorative Dentistry, Faculty of Dentistry, University of Aleppo, Aleppo, Syria; 16 UWA Dental School, The University of Western Australia, Nedlands, Western Australia, Australia; 17 College of Dental Medicine, QU Health, Qatar University, Doha, Qatar; 18 Department of Prosthetic Dental Sciences, King Saud University, Saudi Arabia; 19 Department of Orthodontics, Faculty of Dentistry, Al-Wataniya Private University, Hama, Syria

## Abstract

**Importance:**

Public awareness of oral cancer is crucial for prevention, early detection, and improved survival rates. Knowledge gaps in Middle Eastern and North African countries and areas for public education need to be assessed.

**Objective:**

To evaluate public knowledge and awareness of oral cancer, including risk factors, signs and symptoms, and protective measures, in Middle Eastern and North African countries.

**Design, Setting, and Participants:**

This cross-sectional study was conducted using an open, web-based questionnaire from January to December 2022 across 13 Middle Eastern and North African countries (Saudi Arabia, Syria, Egypt, Jordan, Lebanon, Iraq, Sudan, Morocco, Algeria, Yemen, the United Arab Emirates, Qatar, and Oman). Participants were recruited from the general adult population via social media platforms.

**Main Outcomes and Measures:**

Primary outcomes included knowledge of risk factors, signs and symptoms, and protective measures. Factors associated with good knowledge were evaluated based on age, sex, education level, country income level, smoking status, smokeless tobacco use, and exposure to dental education on oral cancer.

**Results:**

Among 4197 participants (2243 aged 18-30 years [53.4%]; 2372 female [56.5%] and 1825 male [43.5%]), 1559 participants (37.2%) demonstrated good knowledge of oral cancer risk factors, 2028 participants (48.4%) were aware of its signs and symptoms, and 2478 participants (59.1%) recognized associated protective measures. Factors associated with good knowledge across all domains included university education (eg, risk factor knowledge: odds ratio [OR] vs ≤intermediate school, 1.52; 95% CI, 1.20-1.92), no tobacco use (eg, risk factor knowledge: OR for never smoking, 1.44; 95% CI, 1.20-1.74), and receiving education from a dentist (eg, risk factor knowledge: OR, 8.60; 95% CI, 7.33-10.08) at the *P* < .05 significance threshold. Being female (eg, 1491 females [62.9%] vs 987 males [54.1%] with good knowledge) and residing in upper-middle– or high- vs low- and lower-middle–income countries (eg, 1230 of 1891 participants [65.1%] vs 1248 of 2306 participants [54.2%] with good knowledge) were also associated with increased awareness of protective measures at the *P* < .00036 significance threshold with Bonferroni adjustment.

**Conclusions and Relevance:**

This study found a significant lack of oral cancer awareness in Middle Eastern and North African populations, particularly among individuals with lower levels of education and those who used tobacco. Findings also underscore the critical role of dental professionals in improving public knowledge of oral cancer through targeted educational interventions.

## Key Points

**Question:**

What is the level of public awareness and knowledge about oral cancer among individuals in 13 Middle Eastern and North African countries?

**Findings:**

This cross-sectional study of 4197 participants from 13 Middle Eastern and North African countries revealed limited awareness of oral cancer; 37.2% recognized risk factors, 48.4% identified symptoms, and 59.1% understood protective measures. Awareness was higher among individuals who were university educated, did not use tobacco, and were educated by dentists.

**Meaning:**

This study’s findings suggest a critical lack of oral cancer awareness in Middle Eastern and North African countries, underscoring the need for targeted educational efforts by dental professionals and oral health authorities.

## Teaser

This cross-sectional study investigates public knowledge and awareness of oral cancer risk factors, signs and symptoms, and protective measures in Middle Eastern and North African countries.

## Introduction

Oral cancer is a significant global health concern, causing around 529 500 cancer-related deaths annually.^1,2^ The World Health Organization classifies oral and oropharyngeal cancers by location, such as the lip, tongue, and gingiva.^3^ Oral cancer, a major health concern in Southeast Asia, is prevalent due to tobacco, betel quid chewing, smoking, and alcohol consumption. In the US, annual incidences are 51 540 cases.^4,5^ In 2012, the Middle Eastern and North African region had 8928 new cases of oral and oropharyngeal cancer, representing 1.5% of all cancers, with 3573 deaths. The highest incidence rates were in Palestine, Somalia, and Sudan, while Bahrain and Libya had the lowest rates.^6^ These variations underline significant regional differences in oral cancer mortality, potentially influenced by disparities in health care access, public awareness, and lifestyle factors.

Oral cancer risk factors include alcohol, tobacco, human papillomavirus 16 (HPV-16) infection, betel quid, and ultraviolet radiation exposure,^7,8^ while low fruit and vegetable intake may also contribute.^9,10^ Early diagnosis is critical for improving survival rates,^11^ while late diagnosis can negatively impact patient outcomes.^12^

Patient knowledge and professional diagnosis are crucial for improving treatment outcomes, especially in understanding signs and symptoms of oral cancer and unhealthy lifestyle habits.^13^ Public awareness of oral cancer risk factors remains low, with approximately one-quarter of individuals in Maryland in the US recognizing tobacco as a risk factor.^14^

While tobacco use is widely recognized as a major cause of oral cancer, the role of alcohol, particularly when consumed regularly or alongside tobacco, is often overlooked,^15^ despite evidence associating alcohol consumption with an increased risk of developing oral cancer.^16^ Among oral and head-and-neck cancers, 75% of cases are associated with tobacco and alcohol use, with individuals who use both substances at significantly higher risk.^17,18^

HPV-16, particularly prevalent in individuals younger than 50 years, is linked to oral cancer, yet public awareness remains limited.^19^ Perceived oral cancer risk factors include heredity factors, marijuana, HIV, and alcohol in mouthwashes.^19,20^ Additionally, the evaluation of oral cancer risk may be influenced by factors such as melanin levels in patients and dietary habits.

Furthermore, signs and symptoms of oral cancer remain poorly understood by patients.^13,15^ Common symptoms include nonhealing ulcers, visible red or white patches, mouth swelling, and tongue soreness.^21^ Rogers et al^15^ discovered that 1 in 3 patients could accurately identify a nonhealing ulcer as a sign of oral cancer and fewer than 50% of patients were aware of oropharyngeal cancers. Developing public oral health education programs can enhance early detection of oral cancer, thereby improving survival rates.^22^

Public awareness of a disease is undeniably a key factor for its prevention, early detection, and efficient management. Improved awareness is associated with not only enhanced health outcomes but also more cost-effective health care services.^23^ The aim of this study was to assess public knowledge and awareness of oral cancer across various Middle Eastern and North African countries. This included evaluating this population’s understanding of the risk factors, signs, symptoms, and protective measures associated with the disease. Additionally, the study aimed to investigate factors associated with good knowledge about oral cancer.

## Methods

### Study Design and Ethical Approval

This study was designed as a cross-sectional survey using an open, web-based questionnaire following guidelines outlined in the Checklist for Reporting Results of internet E-Surveys (CHERRIES).^24^ Ethical approval was granted by the Ethical Review Board of Prince Sattam bin Abdulaziz University in Al-Kharj, Saudi Arabia. Informed consent was obtained from all participants before they completed the questionnaire.

### Setting and Sample

The study was conducted between January and December 2022 using a convenience sample of the public from 13 countries: Saudi Arabia, Syria, Egypt, Jordan, Lebanon, Iraq, Sudan, Morocco, Algeria, Yemen, the United Arab Emirates, Qatar, and Oman. The sample size was calculated using Open-Source Epidemiologic Statistics for Public Health (OpenEpi) version 3.01 (Emory University). We used 50% for the anticipated frequency as recommended for unknown frequencies, with 2% absolute precision and a 95% CI. The minimum required sample size was 2396 participants. The power analysis for this study was conducted to ensure that the sample size was sufficient to detect a minimum difference of 10% (50% vs 60%) between groups, with an α level of .05. Using G*Power software version 3.1.9.7 (Franz Faul, Universität Kiel, Germany) and the estimated sample size of 2396 participants, the analysis indicated a power of 0.99, which is considered very high and ensures that the study was well-powered to detect the specified differences.

### Questionnaire

The study questionnaire was adapted from previous similar studies.^25,26^ Before the commencement of the study, the questionnaire was reviewed by 6 experts in the field (A.A., F.M.A., S.R., O.K., M.N., and S.A.A) for their feedback and was modified accordingly. Additionally, the questionnaire was piloted among a diverse group of individuals from the 13 countries and adjusted based on their input. A link to the questionnaire was then created using Google Forms (Google) and distributed to potential participants via various social media platforms.

We used a convenience sampling protocol to recruit participants. The survey was translated with the assistance of a certified English translation specialist in medical sciences and distributed in English and Arabic to all participants to ensure transparency. The questionnaire consisted of a cover page and 2 sections. The cover page explained study objectives and invited people to participate, with an emphasis on participant confidentiality and anonymity. Participants took approximately 5 to 6 minutes to complete the survey.

The first section gathered demographic information. In the second section, we assessed participant knowledge of oral cancer, categorized into 3 domains:

1. Risk factors included 6 common risk factors: tobacco (smoking or smokeless forms), alcohol consumption, mouthwash containing alcohol (prolonged use), HPV infection, malnutrition, and a family history of oral cancer.
2. Signs and symptoms listed 6 common indicators of oral cancer, including difficulty chewing or swallowing, persistent mouth sores that do not heal, an abnormal mass or lump in the mouth, a white or red patch inside the mouth, a slow change in voice quality, and weight loss.
3. Protective measures outlined 5 measures to prevent oral cancer: quitting tobacco use, avoiding alcohol consumption, avoiding exposure to secondhand smoke, informing the dentist when dentures do not fit properly, and notifying the dentist about sharp margins in dental restorations to prevent chronic irritation.

Participants were asked to respond to each item by selecting *yes*, *no*, or *do not know*. All questions in the survey were mandatory. Participants were unable to submit the survey unless they had completed all items.

Participants were considered to have *good knowledge* if they correctly answered at least 4 listed items in each knowledge domain, which corresponds to greater than 60% accuracy. This threshold was applied to assess knowledge across domains of risk factors, common signs and symptoms, and protective measures against oral cancer.^27^

### Data Collection

A convenience sample of participants was recruited from 13 Middle Eastern and North African countries through an online survey designed to assess public awareness and knowledge of oral cancer. The survey was disseminated exclusively via social media platforms to ensure broad geographic and demographic reach across target countries participating in this study. Popular platforms, such as Facebook, Instagram, Twitter (now, X), and WhatsApp, were the channels for distribution owing to their widespread use in the region. Participation in the survey was voluntary. The survey was crafted in a manner that encouraged sharing, including purpose and importance.

### Statistical Analysis

Data analysis was conducted using SPSS statistical software version 22.0 (IBM). Descriptive statistics were used to present demographic and other characteristics of participants and their responses to survey questions, categorized by country and demographic variables. The χ^2^ test was performed to assess potential associations of participant demographics and other characteristics with participant knowledge of oral cancer; characteristics included age, sex, education level, country income level, smoking status, smokeless tobacco use, and whether they had received education about oral cancer from a dentist. To control for the increased risk of type I errors due to multiple comparisons, we applied Bonferroni adjustments by dividing the desired significance level (*P* < .05) by the number of tests conducted (140 tests), considering *P* < .00036 as significant. For all comparisons, a 2-sided threshold was used to detect significant differences regardless of direction.

We used logistic regression to identify factors associated with good oral cancer knowledge. Factors investigated included age, sex, education level, country income level, smoking status, smokeless tobacco use, and receipt of oral cancer education from dentists. A *P* value < .05 was considered statistically significant.

## Results

Of 4246 individuals who accessed the survey, 4197 participants (2243 aged 18-30 years [53.4%]; 2372 female [56.5%] and 1825 male [43.5%]) from 13 Middle Eastern and North African countries completed it and were included in the final analysis, resulting in a completion rate of 98.8% among those who accessed the survey. However, the actual response rate cannot be calculated because the total number of people who saw the invitation is unknown. That total is anticipated to be very high, suggesting that the actual response rate was likely very low.

Most respondents (2489 individuals [59.3%]) had a university-level education. The country with the most participants was Saudi Arabia (1213 participants [28.9%]), followed by Syria (480 participants [11.4%]) and Algeria (392 participants [9.3%]). The countries with the fewest participants were the United Arab Emirates, Qatar, and Oman, collectively contributing 104 participants (2.5%). A significant proportion of responses (2306 participants [54.9%]) came from low- and lower-middle–income countries, while 1891 participants (45.1%) were from upper-middle– and high-income countries. Most participants (2687 individuals [64.1%]) reported that they had never smoked. A minority had used smokeless tobacco (703 participants [16.8%]), and 1267 participants (30.2%) had received some form of education on oral cancer from their dentist. Table 1 and the eTable in Supplement 1 present characteristics of the study sample.^28^

**Table 1.** Participant Characteristics

Characteristic	Participants, No. (%) (N = 4197)
Age, y	
18-30	2243 (53.4)
31-45	1372 (32.7)
>45	582 (13.9)
Sex	
Male	1825 (43.5)
Female	2372 (56.5)
Basic education level	
≤Intermediate school	532 (12.7)
Secondary school	1176 (28)
University	2489 (59.3)
Nationality	
Sudan	217 (5.2)
Syria	480 (11.4)
Yemen	128 (3)
Algeria	392 (9.3)
Morocco	337 (8)
Egypt	378 (9)
Lebanon	374 (8.9)
Iraq	214 (5.1)
Jordan	360 (8.6)
Saudi Arabia	1213 (28.9)
Other Gulf state^a^	104 (2.5)
Country income level^b^	
Low and lower middle	2306 (54.9)
Upper middle and high	1891 (45.1)
Smoking status^c^	
Never smoker	2687 (64.1)
Quit or current smoker	1506 (35.9)
Smokeless tobacco status^d^	
Never used	3490 (83.2)
Current or former user	703 (16.8)
Received education on oral cancer from the dentist	1267 (30.2)

^a^ United Arab Emirates, Qatar, and Oman.

^b^ World Bank country classifications by income level for 2021.^28^

^c^ Any kind of smoking, such as cigarettes, cigars, pipes, shisha, e-cigarettes, or other forms.

^d^ Use of any kind of smokeless tobacco, such as chewing tobacco, snuff, snus, or other forms.

Table 2 presents participant knowledge of oral cancer by country and national income level. Overall, 1559 participants (37.2%) demonstrated good knowledge of oral cancer risk factors, with those from Sudan (142 of 217 participants [65.4%]), Iraq (111 of 214 participants [51.9%]), and Yemen (65 of 128 participants [50.8%]) achieving the highest scores. Regarding knowledge of common signs and symptoms, less than half of the study population showed a good level of knowledge (2028 participants [48.4%]), with respondents from Sudan (165 participants [76.0%]) and Yemen (82 participants [64.1%]) again performing best. Awareness of protective measures against oral cancer varied across countries, with Sudanese participants exhibiting the highest level of knowledge (188 participants [86.6%]), while those from Morocco had the lowest level (104 of 337 participants [30.9%]). Overall, 2478 participants (59.1%) demonstrated good knowledge of protective measures against oral cancer.

**Table 2.** Participant Knowledge of Oral Cancer by Country and National Income Level

Knowledge domain	Participants responding *yes*, No. (%)											
	Total (N = 4197)	Low-income country^a^			Lower middle–income country^a^				Upper middle–income country^a^		High-income country^a^	
		Sudan (n = 217)	Syria (n = 480)	Yemen (n = 128)	Algeria (n = 392)	Morocco (n = 337)	Egypt (n = 378)	Lebanon (n = 374)	Iraq (n = 214)	Jordan (n = 360)	Saudi Arabia (n = 1213)	Other Gulf states (n = 104)^b^
Risk factors of oral cancer												
Tobacco	2999 (71.5)	209 (96.3)	364 (75.8)	115 (89.8)	145 (37.0)	196 (58.2)	256 (67.7)	264 (70.8)	128 (59.8)	219 (60.8)	1014 (83.6)	89 (85.6)
Alcohol	3065 (73.0)	191 (88.0)	397 (82.7)	109 (85.2)	203 (51.8)	210 (62.3)	287 (75.9)	258 (69.0)	148 (69.2)	263 (73.1)	916 (75.5)	83 (79.8)
Mouthwash that contains alcohol	2289 (54.5)	150 (69.1)	302 (62.9)	93 (72.7)	106 (27.0)	125 (37.1)	213 (56.3)	183 (48.9)	124 (57.9)	211 (58.6)	725 (59.8)	57 (54.8)
HPV	1174 (28.0)	78 (35.9)	138 (28.8)	30 (23.4)	130 (33.2)	155 (46.0)	104 (27.5)	79 (21.1)	95 (44.4)	59 (16.4)	290 (23.9)	16 (15.4)
Malnutrition	1855 (44.2)	148 (68.2)	231 (48.1)	70 (54.7)	143 (36.5)	128 (38.0)	188 (49.7)	166 (44.4)	110 (51.4)	145 (40.3)	491 (40.5)	35 (33.7)
Family history	1196 (28.5)	123 (56.7)	133 (27.7)	33 (25.8)	109 (27.8)	90 (26.7)	89 (23.5)	115 (30.7)	99 (46.3)	59 (16.4)	324 (26.7)	22 (21.2)
Good knowledge of risk factors^c^	1559 (37.2)	142 (65.4)	198 (41.3)	65 (50.8)	100 (25.5)	102 (30.3)	144 (38.1)	123 (33.0)	111 (51.9)	106 (29.4)	439 (36.2)	29 (27.9)
Common signs and symptoms of oral cancer												
Difficulty chewing or swallowing	2195 (52.3)	165 (76.0)	284 (59.2)	76 (59.4)	140 (35.7)	150 (44.5)	201 (53.2)	191 (51.3)	79 (36.9)	188 (52.4)	667 (55.0)	54 (51.9)
A mouth sore that does not heal	2439 (58.2)	174 (80.2)	337 (70.2)	94 (73.4)	165 (42.1)	152 (45.1)	239 (63.2)	193 (51.9)	81 (37.9)	192 (53.5)	747 (61.6)	65 (62.5)
Abnormal mass or lump in mouth	2462 (58.7)	187 (86.2)	319 (66.5)	103 (80.5)	128 (32.7)	108 (32.0)	225 (59.5)	250 (67.2)	99 (46.3)	196 (54.6)	773 (63.7)	74 (71.2)
White or red patch in mouth	2090 (49.8)	152 (70.0)	277 (57.7)	79 (61.7)	115 (29.3)	133 (39.5)	204 (54.0)	165 (44.4)	82 (38.3)	182 (50.7)	641 (52.8)	60 (57.7)
A slow change in voice quality	1846 (44.0)	160 (73.7)	223 (46.5)	48 (37.5)	97 (24.7)	114 (33.8)	176 (46.6)	143 (38.4)	71 (33.2)	169 (47.1)	593 (48.9)	52 (50.0)
Weight loss	1953 (46.6)	165 (76.0)	254 (52.9)	73 (57.0)	109 (27.8)	100 (29.7)	180 (47.6)	140 (37.6)	75 (35.0)	162 (45.1)	635 (52.3)	60 (57.7)
Good knowledge of symptoms^c^	2028 (48.4)	165 (76.0)	259 (54.0)	82 (64.1)	110 (28.1)	109 (32.3)	197 (52.1)	160 (43.0)	70 (32.7)	171 (47.6)	647 (53.3)	58 (55.8)
Protective measures against oral cancer												
Quit smoking	3150 (75.1)	206 (94.9)	392 (81.7)	123 (96.1)	170 (43.4)	179 (53.1)	284 (75.1)	258 (69.4)	105 (49.1)	263 (73.3)	1079 (89.0)	91 (87.5)
Quit alcohol intake	2939 (70.1)	188 (86.6)	378 (78.8)	110 (85.9)	151 (38.5)	160 (47.5)	272 (72.0)	237 (63.7)	132 (61.7)	257 (71.6)	974 (80.3)	80 (76.9)
Avoid secondhand smoking	2290 (54.6)	166 (76.5)	243 (50.6)	81 (63.3)	133 (33.9)	150 (44.5)	233 (61.6)	241 (64.8)	104 (48.6)	167 (46.5)	705 (58.1)	67 (64.4)
Telling the dentist when dentures do not fit well	2869 (68.4)	203 (93.5)	342 (71.3)	114 (89.1)	148 (37.8)	125 (37.1)	258 (68.3)	267 (71.8)	136 (63.6)	239 (66.6)	963 (79.4)	74 (71.2)
Telling the dentist about sharp margins of restoration	2677 (63.8)	197 (90.8)	313 (65.2)	102 (79.7)	138 (35.2)	115 (34.1)	254 (67.2)	261 (70.2)	129 (60.3)	213 (59.3)	891 (73.5)	64 (61.5)
Good knowledge of protective measures^c^	2478 (59.1)	188 (86.6)	285 (59.4)	99 (77.3)	131 (33.4)	104 (30.9)	231 (61.1)	210 (56.5)	110 (51.4)	209 (58.2)	847 (69.8)	64 (61.5)

Abbreviation: HPV, human papillomavirus.

^a^ Each country was classified according to the World Bank’s income-level classifications for 2021.^28^

^b^ Other Gulf states included the United Arab Emirates, Qatar, and Oman.

^c^ Good knowledge was defined as providing correct answers to 4 or more listed items (>60%).

Never smoking, never using smokeless tobacco, and receiving oral cancer education from a dentist were associated with good knowledge of oral cancer risk factors at the significance threshold of *P* < .00036, while other characteristics were not associated (Table 3 and Table 4). On the same note, being female, having a university education, being a nonsmoker, never using smokeless tobacco, and receiving oral cancer education from a dentist were associated with good knowledge of common signs and symptoms of oral cancer at the significance threshold of *P* < .00036 (Table 3 and Table 4). Regarding protective measures, being female (1491 females [62.9%] vs 987 males [54.1%]; *P* < .00036), having a university education, living in upper-middle– or high- vs low- and lower-middle–income countries (1230 of 1891 participants [65.1%] vs 1248 of 2306 participants [54.2%]; *P* < .00036), never smoking, never using smokeless tobacco, and receiving oral cancer education from a dentist were associated with good knowledge at the *P* < .00036 significance threshold (Table 3 and Table 4).

**Table 3.** Participant Knowledge of Oral Cancer by Participant Demographics

Knowledge domain	Participants responding *yes*, No. (%)											
	Total (N = 4197)	Age, y				Sex			Basic education level			
		18-30 (n = 2243)	31-45 (n = 1372)	>45 (n = 582)	*P* value^a^	Male (n = 1825)	Female (n = 2372)	*P* value^a^	≤Intermediate school (n = 532)	Secondary school (n = 1176)	University (n = 2489)	*P* value^a^
Risk factors of oral cancer												
Tobacco	2999 (71.5)	1599 (71.3)	992 (72.3)	408 (70.1)	.60	1307 (71.7)	1692 (71.3)	.82	276 (52.0)	774 (65.8)	1949 (78.3)	<.00036
Alcohol	3065 (73.0)	1673 (74.6)	977 (71.2)	415 (71.3)	.05	1249 (68.4)	1816 (76.6)	<.00036	324 (60.9)	829 (70.5)	1912 (76.8)	<.00036
Mouthwash that contains alcohol	2289 (54.5)	1338 (59.7)	665 (48.5)	286 (49.1)	<.00036	928 (50.8)	1361 (57.4)	<.00036	248 (46.6)	605 (51.4)	1436 (57.7)	<.00036
HPV	1174 (28.0)	579 (25.8)	393 (28.6)	202 (34.7)	<.00036	559 (30.6)	615 (25.9)	.001	201 (37.8)	325 (27.6)	648 (26.0)	<.00036
Malnutrition	1855 (44.2)	966 (43.1)	618 (45.0)	271 (46.6)	.24	799 (43.8)	1056 (44.5)	.63	229 (43.0)	461 (39.2)	1165 (46.8)	<.00036
Family history	1196 (28.5)	593 (26.4)	410 (29.9)	193 (33.2)	.002	571 (31.3)	625 (26.3)	.0004	198 (37.2)	308 (26.2)	690 (27.7)	<.00036
Good knowledge of risk factors^b^	1559 (37.2)	824 (36.8)	503 (36.7)	232 (39.9)	.35	685 (37.6)	874 (36.8)	.64	190 (35.8)	390 (33.2)	979 (39.3)	.001
Common signs and symptoms of oral cancer												
Difficulty chewing or swallowing	2195 (52.3)	1189 (53.1)	694 (50.6)	312 (53.6)	.28	923 (50.6)	1272 (53.6)	.05	196 (37.0)	561 (47.7)	1438 (57.8)	<.00036
A mouth sore that does not heal	2439 (58.2)	1307 (58.3)	794 (57.9)	338 (58.1)	.96	1003 (55.0)	1436 (60.6)	<.00036	208 (39.2)	581 (49.4)	1650 (66.3)	<.00036
Abnormal mass or lump in mouth	2462 (58.7)	1347 (60.1)	797 (58.1)	318 (54.6)	.05	1007 (55.2)	1455 (61.4)	<.00036	227 (42.8)	564 (48.0)	1671 (67.2)	<.00036
White or red patch in mouth	2090 (49.8)	1144 (51.1)	663 (48.3)	283 (48.6)	.23	853 (46.8)	1237 (52.2)	.001	183 (34.5)	487 (41.4)	1420 (57.1)	<.00036
A slow change in voice quality	1846 (44.0)	957 (42.7)	611 (44.5)	278 (47.8)	.08	795 (43.6)	1051 (44.3)	.64	168 (31.7)	456 (38.8)	1222 (49.1)	<.00036
Weight loss	1953 (46.6)	999 (44.6)	657 (47.9)	297 (51.0)	.01	815 (44.7)	1138 (48.0)	.03	161 (30.4)	439 (37.3)	1353 (54.4)	<.00036
Good knowledge of symptoms^b^	2028 (48.4)	1103 (49.2)	635 (46.3)	290 (49.8)	.17	825 (45.3)	1203 (50.7)	<.00036	164 (30.9)	470 (40.0)	1394 (56.0)	<.00036
Protective measures against oral cancer												
Quit smoking	3150 (75.1)	1747 (78.0)	996 (72.6)	407 (69.9)	<.00036	1306 (71.6)	1844 (77.8)	<.00036	241 (45.5)	772 (65.6)	2137 (85.9)	<.00036
Quit alcohol intake	2939 (70.1)	1621 (72.4)	938 (68.4)	380 (65.3)	.001	1201 (65.9)	1738 (73.3)	<.00036	278 (52.5)	711 (60.5)	1950 (78.4)	<.00036
Avoid secondhand smoking	2290 (54.6)	1210 (54.0)	741 (54.0)	339 (58.2)	.16	1012 (55.5)	1278 (53.9)	.30	216 (40.8)	573 (48.7)	1501 (60.3)	<.00036
Telling dentist when dentures do not fit well	2869 (68.4)	1578 (70.4)	928 (67.6)	363 (62.4)	.001	1150 (63.1)	1719 (72.5)	<.00036	267 (50.4)	657 (55.9)	1945 (78.2)	<.00036
Telling dentist about sharp margins of restoration	2677 (63.8)	1456 (65.0)	877 (63.9)	344 (59.1)	.03	1103 (60.5)	1574 (66.4)	<.00036	267 (50.4)	609 (51.8)	1801 (72.4)	<.00036
Good knowledge of protective measures^b^	2478 (59.1)	1358 (60.6)	799 (58.2)	321 (55.2)	.04	987 (54.1)	1491 (62.9)	<.00036	208 (39.2)	573 (48.7)	1697 (68.2)	<.00036

Abbreviation: HPV, human papillomavirus.

^a^ Significance in this analysis was defined at *P* < .00036 as indicated by χ^2^ statistics with Bonferroni adjustment (.05/140).

^b^ Good knowledge was defined as providing correct answers to 4 or more listed items (>60%).

**Table 4.** Participant Knowledge of Oral Cancer by Other Characteristics

Knowledge domain	Participants responding *yes*, No. (%)												
	Total (N = 4197)	Country income level			Smoking status			Smokeless tobacco status			Received education on oral cancer		
		LLM (n = 2306)	UMH (n = 1891)	*P* value^a^	Never (n = 2687)	Quit or current (n = 1506)	*P* value^a^	Never (n = 3490)	Current or former (n = 703)	*P* value^a^	Yes (n = 1267)	No (n = 2930)	*P* value^a^
Risk factors of oral cancer													
Tobacco	2999 (71.5)	1549 (67.2)	1450 (76.7)	<.00036	2016 (75.0)	981 (65.1)	<.00036	2672 (76.6)	325 (46.2)	<.00036	966 (76.2)	2033 (69.4)	<.00036
Alcohol	3065 (73.0)	1655 (71.8)	1410 (74.6)	.04	2101 (78.2)	962 (63.9)	<.00036	2669 (76.5)	394 (56.0)	<.00036	1022 (80.7)	2043 (69.7)	<.00036
Mouthwash that contains alcohol	2289 (54.5)	1172 (50.8)	1117 (59.1)	<.00036	1622 (60.4)	665 (44.2)	<.00036	2055 (58.9)	232 (33.0)	<.00036	783 (61.8)	1506 (51.4)	<.00036
HPV	1174 (28.0)	714 (31.0)	460 (24.3)	<.00036	705 (26.2)	466 (30.9)	.001	896 (25.7)	275 (39.1)	<.00036	831 (65.6)	343 (11.7)	<.00036
Malnutrition	1855 (44.2)	1074 (46.6)	781 (41.3)	.001	1240 (46.1)	612 (40.6)	.001	1589 (45.5)	263 (37.4)	<.00036	928 (73.2)	927 (31.6)	<.00036
Family history	1196 (28.5)	692 (30.0)	504 (26.7)	.02	718 (26.7)	476 (31.6)	.001	969 (27.8)	225 (32.0)	.02	832 (65.7)	364 (12.4)	<.00036
Good knowledge of risk factors^b^	1559 (37.2)	874 (37.9)	685 (36.2)	.26	1058 (39.4)	499 (33.1)	<.00036	1347 (38.6)	210 (29.9)	<.00036	861 (68.0)	698 (23.8)	<.00036
Common signs and symptoms of oral cancer													
Difficulty chewing or swallowing	2195 (52.3)	1207 (52.4)	988 (52.3)	.94	1499 (55.8)	693 (46.0)	<.00036	1894 (54.3)	298 (42.4)	<.00036	868 (68.5)	1327 (45.3)	<.00036
A mouth sore that does not heal	2439 (58.2)	1354 (58.8)	1085 (57.4)	.37	1666 (62.0)	770 (51.2)	<.00036	2142 (61.4)	294 (41.8)	<.00036	877 (69.2)	1562 (53.4)	<.00036
Abnormal mass or lump in mouth	2462 (58.7)	1320 (57.3)	1142 (60.4)	.04	1672 (62.2)	787 (52.3)	<.00036	2224 (63.8)	235 (33.4)	<.00036	827 (65.3)	1635 (55.9)	<.00036
White or red patch in mouth	2090 (49.8)	1125 (48.8)	965 (51.1)	.15	1451 (54.0)	636 (42.3)	<.00036	1850 (53.0)	237 (33.7)	<.00036	786 (62.0)	1304 (44.6)	<.00036
A slow change in voice quality	1846 (44.0)	961 (41.7)	885 (46.8)	.001	1241 (46.2)	602 (40.0)	<.00036	1611 (46.2)	232 (33.0)	<.00036	717 (56.6)	1129 (38.6)	<.00036
Weight loss	1953 (46.6)	1021 (44.3)	932 (49.3)	.001	1344 (50.0)	606 (40.3)	<.00036	1729 (49.6)	221 (31.4)	<.00036	717 (56.6)	1236 (42.2)	<.00036
Good knowledge of symptoms^b^	2028 (48.4)	1082 (47.0)	946 (50.1)	.05	1415 (52.7)	610 (40.5)	<.00036	1804 (51.7)	221 (31.4)	<.00036	740 (58.4)	1288 (44.0)	<.00036
Protective measures against oral cancer													
Quit smoking	3150 (75.1)	1612 (70.0)	1538 (81.4)	<.00036	2204 (82.1)	943 (62.7)	<.00036	2846 (81.6)	301 (42.8)	<.00036	982 (77.5)	2168 (74.1)	.02
Quit alcohol intake	2939 (70.1)	1496 (64.9)	1443 (76.3)	<.00036	2053 (76.4)	883 (58.7)	<.00036	2647 (75.9)	289 (41.1)	<.00036	983 (77.6)	1956 (66.8)	<.00036
Avoid secondhand smoking	2290 (54.6)	1247 (54.1)	1043 (55.2)	.49	1544 (57.5)	743 (49.4)	<.00036	2023 (58.0)	264 (37.6)	<.00036	860 (67.9)	1430 (48.9)	<.00036
Telling dentist when dentures do not fit well	2869 (68.4)	1457 (63.2)	1412 (74.7)	<.00036	1991 (74.1)	876 (58.2)	<.00036	2629 (75.4)	238 (33.9)	<.00036	924 (72.9)	1945 (66.5)	<.00036
Telling dentist about sharp margins of restoration	2677 (63.8)	1380 (59.9)	1297 (68.6)	<.00036	1847 (68.8)	828 (55.0)	<.00036	2443 (70.0)	232 (33.0)	<.00036	906 (71.5)	1771 (60.5)	<.00036
Good knowledge of protective measures^b^	2478 (59.1)	1248 (54.2)	1230 (65.1)	<.00036	1767 (65.8)	709 (47.1)	<.00036	2270 (65.1)	206 (29.3)	<.00036	824 (65.0)	1654 (56.5)	<.00036

Abbreviations: HPV, human papillomavirus; LLM, low and lower middle; UMH, upper middle and high.

^a^ Significance in this analysis was defined at *P* < .00036 as indicated by χ^2^ statistics with Bonferroni adjustment (.05/140).

^b^ Good knowledge was defined as providing correct answers to 4 or more listed items (>60%).

The multivariate logistic regression analysis in Table 5 investigated factors associated with good knowledge of oral cancer, including awareness of risk factors, recognition of common signs and symptoms, and understanding of protective measures. Associated factors at the *P* < .05 threshold were having a university education (eg, risk factor knowledge: odds ratio [OR] vs ≤intermediate school, 1.52; 95% CI, 1.20-1.92), never smoking (eg, risk factor knowledge: OR, 1.44; 95% CI, 1.20-1.74), never using smokeless tobacco (eg, risk factor knowledge: 1.71; 95% CI, 1.34-2.20), and receiving oral cancer education from a dentist (eg, risk factor knowledge: OR, 8.60; 95% CI, 7.33-10.08).

**Table 5.** Factors Associated With Good Knowledge of Oral Cancer

Factor	Good knowledge, OR (95% CI)^a^		
	Risk factors of oral cancer	Common signs and symptoms of oral cancer	Protective measures against oral cancer
Age, y			
18-30	1 [Reference]	1 [Reference]	1 [Reference]
31-45	1.02 (0.87-1.20)	0.92 (0.80-1.06)	1.01 (0.87-1.17)
>45	1.07 (0.86-1.33)	1.17 (0.96-1.42)	1.03 (0.84-1.27)
Sex			
Male	1 [Reference]	1 [Reference]	1 [Reference]
Female	1.02 (0.88-1.20)	1.14 (0.99-1.31)	1.16 (0.99-1.34)
Basic education level			
≤Intermediate school	1 [Reference]	1 [Reference]	1 [Reference]
Secondary school	1.01 (0.79-1.30)	1.55 (1.23-1.94)	1.42 (1.13-1.77)
University	1.52 (1.20-1.92)	2.86 (2.31-3.55)	2.74 (2.22-3.39)
Country income level			
Low and lower middle	1 [Reference]	1 [Reference]	1 [Reference]
Upper middle and high	0.93 (0.81-1.08)	1.08 (0.95-1.23)	1.44 (1.26-1.65)
Smoking status^b^			
Quit or current smoker	1 [Reference]	1 [Reference]	1 [Reference]
Never smoker	1.44 (1.20-1.74)	1.25 (1.06-1.47)	1.25 (1.05-1.48)
Smokeless tobacco status^c^			
Current or former user	1 [Reference]	1 [Reference]	1 [Reference]
Never used	1.71 (1.34-2.20)	1.66 (1.33-2.07)	3.00 (2.40-3.76)
Received education on oral cancer from the dentist			
No	1 [Reference]	1 [Reference]	1 [Reference]
Yes	8.60 (7.33-10.08)	2.31 (2.00-2.67)	2.10 (1.80-2.45)

Abbreviation: OR, odds ratio.

^a^ ORs and 95% CIs were calculated by a multivariate binary logistic model. Significance in this analysis was defined at *P* < .05.

^b^ Any kind of smoking, such as cigarettes, cigars, pipes, shisha, e-cigarettes, or other forms.

^c^ Use of any kind of smokeless tobacco, such as chewing tobacco, snuff, snus, or other forms.

## Discussion

This cross-sectional study assessed knowledge and awareness of oral cancer among more than 4000 participants enrolled from 13 Middle Eastern and North African countries. We found that fewer than half of participants could identify symptoms, approximately one-third knew risk factors, and slightly more than half understood protective measures. Awareness of oral cancer, its risk factors, signs and symptoms, and protective measures can encourage early intervention, which is associated with improved survival rates.^25,29^ By shedding light on the current state of oral cancer awareness, our study lays a vital foundation for future public health interventions that could have a lasting impact on lives of individuals across the Middle East and North Africa.

Spanning 22 countries over 13 million square kilometers in the Middle Eastern and North African region, the Arab world is home to more than 440 million people, each with distinct cultural, historical, and health care contexts. This diversity presents challenges and opportunities in formulating public health initiatives tailored to the region’s unique needs.

Knowledge of oral cancer risk factors, signs and symptoms, and protective measures varied across the study population, with awareness levels of 37.2%, 48.4%, and 59.1%, respectively. These findings reflect a general lack of adequate knowledge, particularly regarding risk factors and signs and symptoms, consistent with previous studies.^30,31^ Public education programs are needed to increase awareness and improve knowledge about oral cancer in Middle Eastern and North African countries. Notably, participants from Sudan and Yemen showed better general knowledge compared with participants from other countries, aligning with studies from Yemen^32^ and Sudan,^33^ where 71.5% and 66.6% of respondents, respectively, were aware of oral cancer.

Participants with a university education demonstrated higher knowledge scores regarding oral cancer compared with participants with lower education levels, consistent with previous studies showing that knowledge increases with higher educational attainment.^34,35^ These results underscore the importance of community engagement, ongoing education, and early information dissemination as key strategies for enhancing public awareness of oral cancer.

While approximately one-third of participants had good overall knowledge of risk factors for oral cancer, roughly three-quarters of participants correctly identified tobacco and alcohol as risk factors. This is consistent with findings from earlier studies^36,37^ and indicates that, for example, antitobacco campaigns may have been successful at emphasizing the harmful effects of smoking. Unfortunately, our results revealed insufficient knowledge regarding HPV as a risk factor for oral cancer, with 28.0% recognizing its association with the disease. Notably, individuals who received education from dentists demonstrated significantly higher awareness of HPV. Given that younger individuals, residents of upper-middle– and high-income countries, and nonusers of smokeless tobacco had lower levels of knowledge, public awareness campaigns regarding harms of HPV (and potentially promoting the HPV vaccine if available) could be targeted at these groups, with active involvement from dental professionals.

It is important to note that the low level of awareness identified in this study regarding signs and symptoms of oral cancer among the study population may contribute to late clinical presentation and, consequently, lower survival rates.^4,5^ This finding emphasizes the urgent need for increased attention and targeted interventions.

Sex and economic disparities emerged as factors associated with awareness of oral cancer in this study. Female participants demonstrated better knowledge of signs, symptoms, and protective measures compared with male participants, likely owing to greater engagement with educational programs via media and higher general health awareness,^32^ aligning with findings from previous studies.^37,38^ Awareness of protective measures also varied by economic status, with higher awareness levels observed in upper-middle– and high-income countries compared with lower-income settings. This aligns with findings from Haley et al,^39^ who highlighted the potential role of expanded insurance coverage in improving oral cancer detection and early screening in low-resource settings. Addressing these disparities through targeted awareness campaigns in the Middle Eastern and North African world tailored to sex- and country-specific needs could be associated with enhanced early diagnosis and survival rates.^40^

Our study considered smoking a risk factor for oral cancer and smoking cessation a protective measure. It can be argued that the public may lack a clear understanding of the connection between tobacco use as a risk factor and the protective benefits of quitting smoking, highlighting a significant knowledge gap. This suggests that public health messaging in the region may have focused more on the dangers of smoking than the benefits of cessation. Misconceptions, such as the belief that smoking-related harm is irreversible, additionally emphasize the need for improved education on the health benefits of quitting, particularly in reducing the risk of oral cancer.

A notable finding was that individuals who had never smoked or used smokeless tobacco demonstrated significantly better knowledge of oral cancer compared with current or former smokers, consistent with prior study findings. Haddad et al^41^ found that individuals who did not smoke were more aware of smoking risks and held more positive attitudes toward smoking bans. Similar findings have been reported in several previous studies.^42,43^ This could reflect the impact of antismoking campaigns^41^ that highlight the harmful effects of smoking and its links to noncommunicable diseases, such as cardiovascular and respiratory conditions, bladder cancer, and oral cancer.^44^ These findings emphasize the need for comprehensive smoking-cessation strategies that enhance public knowledge, attitudes, and behaviors, with implications for reducing oral cancer risk.

Interestingly, participants who received education from their dentist exhibited significantly greater knowledge of oral cancer risk factors, signs, symptoms, and preventive measures. These findings support those of Razavi et al,^45^ who noted that most patients (90%) expected their dentists to inform them about smoking’s harmful effects and were more likely to quit smoking if advised by their dentist. This highlights the impactful role dentists can play in smoking cessation efforts. However, our findings contrast with earlier studies suggesting that dentists often play a secondary role in patient education.^8,44^ For example, Horowitz et al^36^ found that 25% of dentists strongly agreed that they were adequately trained to perform oral cancer screenings. This gap highlights the critical need for enhanced training programs, empowering dental professionals to serve as primary educators and advocates for oral cancer prevention and early detection.

Regression analysis identified key factors associated with knowledge of oral cancer. A key finding was that smoking behavior in the Middle Eastern and North African region was strongly associated with knowledge of oral cancer, including its manifestations, risk factors, and protective measures. This underscores the need for targeted awareness campaigns focusing on individuals who use tobacco, aiming to promote cessation and prevent oral cancer progression. Additionally, higher education levels and dentist-provided education were associated with greater knowledge, highlighting the importance of engaging less-educated populations through tailored educational interventions. Dentists play a critical role in disseminating information and mitigating the fatal consequences of oral cancer. Overall, study findings support the development of standardized oral care guidelines and emphasize the need for comprehensive strategies that engage health care professionals and the public, particularly individuals who use tobacco and those with limited education.

This study provides valuable baseline information on oral cancer awareness and knowledge among the public in 13 Middle Eastern and North African countries. The findings may inform health planners and guide awareness campaigns across the region. Future research should strive for a more representative sample of the entire Middle Eastern and North African population, with a broader inclusion of socioeconomic and demographic variables. Qualitative research may also offer deeper insights in this regard.

### Strengths and Limitations

While the relatively large sample size, high completion rate, and participation from 13 Middle Eastern and North African countries were notable strengths of this study, several limitations must be acknowledged. First, results may not be fully generalizable to the entire Middle Eastern and North African population due to the absence of participation from 9 of 22 countries in the region. Additionally, the variation and imbalance in the number of participants across surveyed countries may impact the robustness and representativeness of findings. Another limitation is the use of close-ended questions to assess participant knowledge. Some questions may have been leading given that they were framed in a way that could suggest *yes* as the correct answer. This may have resulted in response bias, with participants potentially agreeing with statements rather than expressing disagreement, leading to an inflated perception of knowledge. Additionally, the study did not assess the frequency of participant visits to the dentist or the extent of information provided during patient education sessions, which could influence knowledge levels. Furthermore, key social and demographic variables were not collected, limiting our understanding of how these factors were associated with knowledge and awareness of oral cancer. Additionally, the targeted group of individuals with lower educational levels, who may benefit most from oral cancer awareness and prevention efforts, may be underrepresented. These individuals may have limited access to social media or may not be as engaged in online health discussions, which can lead to a lack of perspective on the findings.

## Conclusions

This cross-sectional study highlights a concerning lack of awareness about oral cancer among the Middle Eastern and North African public, particularly among individuals with lower educational levels and current or former users of tobacco. Findings underscore the crucial role of dentists in increasing awareness and educating the public about oral cancer. These results may guide oral and public health planners across the region in developing targeted strategies to enhance public awareness and promote early detection of oral cancer.

## Notes

Supplement 1. **eTable.** Sample characteristics by demographics and country **eAppendix.** Study questionnaire

Supplement 2.
Data Sharing Statement
