# Supplementary material for: Public Awareness and Knowledge of Oral Cancer in 13 Middle Eastern and North African Countries
Source: JAMA Netw Open. 2025 Mar 6;8(3):e250522. doi: 10.1001/jamanetworkopen.2025.0522 (PMC11886726; doi:10.1001/jamanetworkopen.2025.0522)
Supplement: Supplement 1. — eTable. Sample characteristics by demographics and country eAppendix. Study questionnaire [file jamanetwopen-e250522-s001.pdf]

Supplemental Online Content

Nassani MZ, Alsalhani A, Alali FM, et al. Public awareness and knowledge of oral cancer in 13 Middle Eastern and North African countries. *JAMA Netw Open*. 2025;8(3):e250522. doi:10.1001/jamanetworkopen.2025.0522

eTable. Sample characteristics by demographics and country

eAppendix. Study questionnaire

This supplemental material has been provided by the authors to give readers additional information about their work.

© 2025 Nassani MZ et al. *JAMA Network Open*.

eTable. Sample characteristics by demographics and country

Demographics	Sample n=4197	Low-Income			Lower middle income				Upper-middle income		High income	
		Sudan n=217	Syria n=480	Yemen n=128	Algeria n=392	Morocco n=337	Egypt n=378	Lebanon n=374	Iraq n=214	Jordan n=360	Saudi Arabia n=1213	Other Gulf States n=104
**Age**												
18-30 years	53.4%	24.9%	51.5%	76.6%	36.7%	48.1%	64.6%	60.7%	35.0%	67.8%	59.2%	28.8%
31-45 years	32.7%	27.6%	38.3%	18.8%	47.7%	33.8%	25.7%	31.0%	45.3%	20.8%	30.9%	41.3%
> 45 years	13.9%	47.5%	10.2%	4.7%	15.6%	18.1%	9.8%	8.3%	19.6%	11.4%	9.9%	29.8%
**Gender**												
Male	43.5%	70.5%	38.1%	37.5%	46.2%	46.6%	36.0%	28.3%	54.7%	31.4%	46.7%	61.5%
Female	56.5%	29.5%	61.9%	62.5%	53.8%	53.4%	64.0%	71.7%	45.3%	68.6%	53.3%	38.5%
**Basic Education Level**												
Intermediate school or less	12.7%	3.7%	10.6%	3.9%	20.7%	14.2%	12.7%	17.4%	57.0%	14.2%	4.2%	1.9%
Secondary school	28.0%	10.6%	23.1%	28.1%	45.2%	37.1%	23.5%	28.1%	20.1%	18.3%	31.0%	24.0%
University	59.3%	85.7%	66.3%	68.0%	34.2%	48.7%	63.8%	54.5%	22.9%	67.5%	64.8%	74.0%
**Smoking Status***												
Never Smoker	64.1%	53.9%	73.1%	82.0%	46.7%	52.5%	76.2%	46.9%	54.7%	63.1%	72.3%	69.2%
Quit or Current Smoker	35.9%	46.1%	26.9%	18.0%	53.3%	47.5%	23.8%	53.1%	45.3%	36.9%	27.7%	30.8%
**Smokeless tobacco status^**												
Never used	83.2%	91.2%	89.8%	91.4%	50.0%	54.6%	89.7%	88.2%	66.8%	82.2%	96.0%	92.3%
Current or Ex-user	16.8%	8.8%	10.2%	8.6%	50.0%	45.4%	10.3%	11.8%	33.2%	17.8%	4.0%	7.7%
**Education about oral cancer**												
Received education on oral cancer from the dentist	30.2%	47.0%	24.0%	27.3%	33.4%	48.1%	29.1%	24.9%	47.2%	18.9%	27.3%	18.3%

Table super-header: Sample characteristics by demographics and country/ Data are presented as (%) — Country

***:** use of any kind of smoking such as cigarettes, cigars, pipes, shisha, E-cigarettes, or other forms

^: use of any kind of smokeless tobacco such as chewing tobacco, snuff, snus, or other forms.

- Other Gulf States: United Arab Emirates, Qatar, and Oman
- Each country is classified according to the World Bank's income-level classifications for 2021. Available online at: https://blogs.worldbank.org/en/opendata/world-bank-country-classifications-by-income-level-for-2024-2025 (accessed December 16, 2024).

© 2025 Nassani MZ et al. *JAMA Network Open*.

Patients Awareness of Mouth Cancer

استبانة عن وعي المرضى بسرطان الفم

Our patients need to be aware of mouth cancer risks. Your answers will help us to improve the information and care that we provide relating to mouth cancer. This survey is voluntary, and it will take about 5 minutes to finish. All information will remain private.

من المهم أن يكون مرضانا على دراية بمخاطر الإصابة بسرطان الفم. ستساعدنا إجاباتك على تحسين المعلومات والرعاية التي نقدمها فيما يتعلق بسرطان الفم. هذا الاستطلاع تطوعي وسيستغرق حوالي 5 دقائق للانتهاء. ستبقى جميع المعلومات خاصة.

Section 1. Demographic data: القسم الأول. البيانات الشخصية:

1. How old are you?		كم عمرك؟
1	18-30 years	من 18 – 30 سنة
2	31-45 years	من 31 – 45 سنة
3	Over 45 years	فوق 45 سنة

2. What is your gender?		ما هو جنسك؟
1	Male	ذكر
2	Female	أنثى

3. What is the highest degree or level of school you have completed?		ما هي أعلى درجة تعليمية حصلت عليها
	Intermediate school or less	مستوى إعدادي أو أقل
	Secondary school	مستوى ثانوي
	University	مستوى جامعي

4. Kindly choose your current country of residency.		الرجاء اختيار مكان اقامتك الحالية
1	Sudan	السودان
2	Syria	سوريا
3	Yemen	اليمن
4	Algeria	الجزائر
5	Morocco	المغرب
6	Egypt	مصر
7	Lebanon	لبنان
8	Iraq	العراق
9	Jordan	الأردن

© 2025 Nassani MZ et al. *JAMA Network Open*.

10	Saudi Arabia	المملكة العربية السعودية
11	UAE	الإمارات العربية المتحدة
12	Qatar	قطر
13	Oman	عمان

**5. What is your current smoking status?** (use of any kind of smoking such as cigarettes, cigars, pipes, shisha, E-cigarettes, or other forms) **Select the option that best describes you:**		**ما هي حالتك الحالية فيما يتعلق بالتدخين؟** (استخدام أي نوع من التدخين مثل السجائر، السيجار، الغليون، الشيشة، السجائر الإلكترونية، أو أشكال أخرى) **اختر الخيار الذي يصفك بشكل أفضل**
1	Never smoking	لم أدخن مطلقا
2	Quit or current Smoker	مدخن حالي او مدخن سابق

**6. What is your current smokeless tobacco use status?** (use of any kind of smokeless tobacco such as chewing tobacco, snuff, snus, or other forms) **Select the option that best describes you:**		**ما هي حالتك الحالية فيما يتعلق مع مضغ التبغ ؟** (استخدام أي نوع من التبغ غير المدخن مثل التبغ الممضوغ، السعوط، السنوس، أو أشكال أخرى) **اختر الخيار الذي يصفك بشكل أفضل**
1	Never use smokeless tobacco	لم اقوم بمضغ التبغ مطلقا
2	Current or Ex-user of smokeless tobacco	أقوم بمضغ التبغ أو استخدمته في الماضيو توقفت عن اسنخدامه

**7. Has your doctor ever educated you about the risks of mouth cancer?**		8. هل قام طبيبك بتثقيفك حول مخاطر سرطان الفم؟
1	Yes	نعم
2	No	لا

Section 2. Knowledge of risk factors of oral cancer

القسم الثاني. المعرفة عن عوامل الخطورة بسرطان الفم

**Select the (yes/ No) answer to the following question:** **أجب (نعم / لا) عن الأسئلة التالية:**	**Yes نعم**	**No لا**
**8. Do you think that tobacco use is considered a risk factor for oral cancer?** **هل تعتقد ان استخدام منتجات التبغ يرفع الاصابة بسرطان الفم**		
**9. Do you think that drinking alcohol is considered a risk factor for oral cancer?**		

© 2025 Nassani MZ et al. *JAMA Network Open*.

هل تعتقد ان شرب الكحول يرفع الاصابة بسرطان الفم		
10. Do you think that the use of mouthwash containing a variable percentage of alcohol increases the risk factor of oral cancer?<br>هل تعتقد أن بعض مركبات غسول الفم الكحولية تزيد الاصابة بسرطان الفم ؟		
11. Have you known that Human papillomavirus (HPV) can cause mouth cancer? هل تعلم أن فيروس الحليمي البشري يسبب سرطان الفم ؟		
12. Do you know that malnutrition or long-term inappropriate diet can Increase the chance of mouth cancer? هل تعلم أن سوء التغذية أو الالتزام بنظام صحي غير لائق يرفع الاصابة بسرطان الفم ؟		
13. Has your dentist told you that a family history of oral cancer increases the chance of mouth cancer? هل أخبرك طبيب الأسنان سابقا ان وجود إصابة عائلية لسرطان الفم يرفع نسبة إصابتك بسرطان الفم ؟		

Section 3. Knowledge of common signs and symptoms of oral cancer:

القسم الثالث. المعرفة عن الأعراض والعلامات السريرية:

14. Which of the following may be a sign of mouth cancer? ما هي أعراض سرطان الفم			
	Yes<br>نعم	No<br>لا	I do not<br>لآ أعلم
Difficulty chewing/swallowing صعوبة في المضغ / البلع			
Mouth sore that does not heal قرحة فموية مزمنة			
Abnormal mass/lump in mouth كتلة غير طبيعية في الفم			
White/red patch in mouth بقع بيضاء أو حمراء في الفم			
Slow change in voice quality تغير بطيء في نوعية الصوت			
Weight loss نقص وزن			

Section 4. Knowledge of protective measures against oral cancer

القسم الرابع. المعرفة عن طرق الوقاية من سرطان الفم

15. Which of the following actions may prevent mouth cancer?<br>أيٌ من الممارسات التالية تمنع حدوث سرطان الفم			
	Yes	No	I do not
Quit tobacco use إيقاف استعمال التبغ			
Quit alcohol use أيقاف شرب الكحول			
Avoid contact with secondhand smoke تقليل الاختلاط مع شخص مدخن			
Telling your dentist when your denture(s) do not fit well إعلام طبيب أسنانك عند ملاحظتك لأي مشكلة في جهازك المتحرك			
Telling your dentist with sharp margins of restoration إعلام طبيب أسنانك عند وجود أي طرف حاد أو مؤذي في حواف الحشوات السنية			

Thank you for your input.

© 2025 Nassani MZ et al. *JAMA Network Open*.
