# Supplementary material for: Public Awareness and Knowledge of Oral Cancer in 13 Middle Eastern and North African Countries
Source: JAMA Netw Open. 2025 Mar 6;8(3):e250522. doi: 10.1001/jamanetworkopen.2025.0522 (PMC11886726; doi:10.1001/jamanetworkopen.2025.0522)
Supplement: Supplement 2. — Data Sharing Statement [file jamanetwopen-e250522-s002.pdf]

Data Sharing Statement

Nassani. Public Awareness and Knowledge of Oral Cancer in 13 Middle Eastern and North African Countries. *JAMA Netw Open*. Published online March 6, 2025. doi:10.1001/jamanetworkopen.2025.0522

Data
Data available: No

Additional Information
Explanation for why data not available: The data supporting the findings of this study are available from the corresponding author upon request.
